# Gamete production patterns, ploidy, and population genetics reveal evolutionary significant units in hybrid water frogs (*Pelophylax esculentus*)

**DOI:** 10.1002/ece3.687

**Published:** 2013-07-30

**Authors:** Nicolas B M Pruvost, Alexandra Hoffmann, Heinz-Ulrich Reyer

**Affiliations:** Institute of Evolutionary Biology and Environmental Studies, University of ZurichWinterthurerstrasse 190, 8057, Zurich, Switzerland

**Keywords:** Breeding system, hybrid speciation, hybridization, hybridogenesis, polyploidy

## Abstract

The European water frog *Pelophylax esculentus* is a natural hybrid between *P. lessonae* (genotype LL) and *P. ridibundus* (RR). It reproduces through hybridogenesis, eliminating one parental genome from its germline and producing gametes containing the genome of the other parental species. According to previous studies, this elimination and transmission pattern is very diverse. In mixed populations, where only diploid hybrids (LR) live in sympatry and mate with one or both parental species, the excluded genome varies among regions, and the remaining genome is transmitted clonally to haploid gametes. In all-hybrid populations consisting of diploid (LR) and triploid (LLR and/or LRR) frogs, diploid individuals also produce gametes clonally (1n in males, 2n in females), whereas triploids eliminate the genome they have in single copy and produce haploid gametes containing the recombined other genome. However, here, too, regional differences seem to exist, and some triploids have been reported to produce diploid gametes. In order to systematically study such regional and genotype differences in gamete production, their potential origin, and their consequences for the breeding system, we sampled frogs from five populations in three European countries, performed crossing experiments, and investigated the genetic variation through microsatellite analysis. For four populations, one in Poland, two in Germany, and one in Slovakia, our results confirmed the elimination and transmission pattern described above. In one Slovakian population, however, we found a totally different pattern. Here, triploid males (LLR) produce sperm with a clonally transmitted diploid LL genome, rather than a haploid recombined L genome, and LR females clonally produce haploid R eggs, rather than diploid LR eggs. These differences among the populations in gamete production go along with differences in genomotype composition, breeding system (i.e., the way triploids are produced), and genetic variation. These differences are strong evidence for a polyphyletic origin of triploids. Moreover, our findings shed light on the evolutionary potential inherent to the *P. esculentus* complex, where rare events due to untypical gametogenetic processes can lead to the raise, the perpetuation, and the dispersion of new evolutionary significant lineages which may also deserve special conservation measures.

## Introduction

Fertile taxa of hybrid origin are pushing the biological species concept to its limits (Dobzhansky 1937; Mayr 1942; Mallet 2008). By allowing genetic interactions between well-defined and differentiated taxa, hybrids are challenging the most acknowledged mode of speciation by divergence followed by reproductive isolation, and they allow scrutinizing the consequences of gene transfer between “good species.” Hence, hybrids constitute biological models of high interest in evolutionary biology and represent valuable material for the ongoing debate on the definition of the nature of species (i.e., whether they are real entities or just arbitrary constructs of the human mind) and on the process of speciation (Mallet 2001; Coyne and Orr 2004; Abbott et al. 2008).

Secondary contact of diverged genetic entities can lead to hybridization when it happens before effective premating barriers have developed. However, failure in segregation of chromosomes from different species often leads to a tremendous fitness decrease in the hybrids’ offspring, ranging from zygotic mortality to inviability or infertility. Some hybrid taxa have escaped the genetic incompatibilities and the resulting detrimental effects on fitness by abandoning normal meiosis. In vertebrates, they have shifted from sexual to clonal genome transmission and adopted one of the following three reproductive modes:

- In parthenogenesis, offspring develop from unreduced eggs without any male input.
- In gynogenesis such unreduced egg need the contact with sperm to trigger the development, but do not incorporate the paternal genetic material.
- In hybridogenesis (Schultz 1969), one of the parental genomes is excluded during the first steps of meiosis, followed by the production of clonal gametes containing the other parental genome. By living in sympatry and mating with the parental species, whose genome has been excluded, hybridity is reestablished and thus a hemiclonal hybrid line perpetuated. Such a reproductive mode has been shown to exist and be quite stable in natural animal populations of insects (*Bacillus*, Mantovani and Scali 1992), fishes (*Squalius*, Carmona et al. 1997 and *Poeciliopsis*, Schultz 1966), and anurans (*Pelophylax*, Berger 1968).

Where problems of chromosome pairing during gametogenesis lead to occasional failure or regular circumvention of chromosome segregation, and hence the production of unreduced gametes, an increase in the ploidy level of the offspring can result (Vrijenhoek 1989; Ramsey and Schemske 1998). Thus, there is a link between hybridization, asexual reproduction, and polyploidization which creates genetic systems with the potential for hybrid speciation through allopolyploidization (Choleva et al. 2012).

The probability of establishing an independently evolving polyploid hybrid lineage can be expected to increase with (1) the rate and type (in terms of genomic composition) at which unreduced gametes are produced, (2) the likelihood that they will fuse, (3) the viability and fertility of the resulting allopolyploid offspring, and (4) the reproductive isolation of such offspring from its parental species and their competitive ability. Chances of establishing a stable and self-perpetuating polyploid lineage are expected to be highest for even ploidy (e.g., tetraploidization) because it allows biparental reproduction with normal meiosis. It has been shown, however, that triploid forms-producing diploid gametes in one sex and haploid ones in the other sex can act as a stepping stone toward tetraploidization (triploid bridge; Ramsey and Schemske 1998; Mable 2004; Cunha et al. 2008). Moreover, as hybrids are often capable of occupying habitats beyond the limits of their diploid progenitors (Endler 1973; Moore 1977; Arnold 1997), we can expect that if such hybrids manage to produce the necessary gamete types, they can replace populations of their parental species. Thus, under certain genetic and ecological conditions hybrids can become evolutionary independent units.

The evolutionary impact of hybridization and polyploidy has been well demonstrated among plant species (Stebbins 1950; Grant 1971; Rieseberg 1997), but examples from the animal kingdom are scarce, especially when it comes to vertebrates (Arnold 1997; Mallet 2008; Schwenk et al. 2008). In this study, we address the first above mentioned condition for polyploidy, that is, the types of gametes produced by different genomotypes, in anuran populations containing triploid individuals.

### The *Pelophylax esculentus* complex

An excellent model system for investigating the evolutionary impact of polyploid hybrids and the associated shift from sexual to clonal genome transmissions is provided by Palearctic water frogs of the *Pelophylax esculentus* complex (formerly genus *Rana* until Frost et al. 2006). The complex is composed of two parental species, the pool frog *P. lessonae* (Camerano 1882) and the marsh frog *P. ridibundus* (Pallas 1771), and their interspecific hybrid *P. esculentus* (Linnaeus 1758), the edible frog. Hybrids of both sexes overcome meiotic pairing problems of *lessonae* (L) and *ridibundus* (R) chromosomes by excluding one of the parental genomes during the first division of gametogenesis and transmitting only the other genome (hybridogenesis; Schultz 1969; Graf and Müller 1979). The original hybrid status is restored by mating with a partner that provides the eliminated genome.

This basic pattern comes in three major variations. In the most widespread case, diploid hybrids (genotype LR) exclude the L genome, produce haploid gametes with a clonal R genome, and restore hybridity by mating with *P. lessonae* (genotype LL). Thus, they are forced to live in sympatry with at least this parental species, thus constituting so-called LE-systems. In the mirror system, named RE-system, the R genome is excluded, and the L genome transmitted, which forces *P. esculentus* to live and mate with *P. ridibundus* (genotype RR) to perpetuate its hybrid line. There is a tendency for LE-systems to be more frequent in Western Europe and RE-systems to dominate in Eastern Europe, but numerous exceptions exist. What generates these two breeding systems remains a puzzle because the exact mechanisms of genome exclusion are still not known; nor are the factors that determine which parental genome is inducing, respectively resisting, exclusions under what conditions. In both systems, however, the hybrids are acting as sexual parasites of a parental host species.

In the northern parts of the species’ range, especially around the Baltic Sea, a third breeding system type exists: the EE-system (Plötner 2005; Christiansen 2009; Arioli et al. 2010; Jakob et al. 2010). Here, populations consist of hybrids only, with no parental species occurring in the surrounding area. Those all-hybrid populations are composed of diploid hybrids (genome LR) and triploids with the LLR and/or LRR genome composition. In this system, diploid females usually produce diploid LR gametes, whereas triploids produce haploid gametes containing the recombined genome of the type they have in double dose, that is, L in LLR frogs and R in LRR (Christiansen 2009; Christiansen and Reyer 2009). This mechanism has been termed “meiotic hybridogenesis” (Alves et al. 1998; Morishima et al. 2008). The production of these three gamete types allows the generation and persistence of the all-hybrid populations. Differences in gamete production, rather than variation in ecological selection regimes, seem to explain why the proportions of LR, LLR, and LRR frogs differ among ponds (gamete pattern hypothesis versus selection hypothesis; Christiansen et al. 2010; Embrechts and Reyer 2012).

These findings are based on intensive studies of all-hybrid populations in Denmark and southern Sweden (Christiansen and Reyer 2009; Arioli et al. 2010; Jakob et al. 2010). However, triploid hybrids have also been reported for several populations south of the Baltic Sea and in Central Europe, where they occur either with only diploid hybrids or with diploids and one or both parental species together (Berger 1988a; Tunner and Heppich-Tunner 1992; Mikulíček and Kotlík 2001; Plötner 2005).

So far, detailed water frog studies have focused on populations within a limited geographic area and on a particular system, that is, either LE- or RE-system where diploid hybrids live and mate with a parental species or EE-system where diploid and triploid hybrids co-occur in the absence of any parental species. However, given the marked regional differences among populations, we felt that a large-scale comparative study between populations with and without triploid individuals was needed. The purpose of our study was to systematically investigate regional and genotype differences in gamete production, their consequences for the breeding system, and whether triploid frogs are of mono- or polyphyletic origin. For this study, we sampled five European populations from four different river basins and performed two different analyses. First, we conducted crossing experiments to analyze the types of gametes produced by the different hybrid genomotypes, that is, the genomic constitution in terms of the number and origin of the constitutive genomes (Lowcock 1994). Second, we used microsatellite analysis to calculate population genetics parameters, such as expected heterozygosity (H_e_, Nei 1978) and fixation index (*F*_ST_, Weir and Cockerham 1984). Together, the two approaches allowed us to infer the breeding systems and their similarities, respectively difference, in different populations. Based on our results, we then discuss possible origins of the systems, the evolutionary potential they carry and their conservation value.

## Material and Methods

### Populations

**Figure 1:**
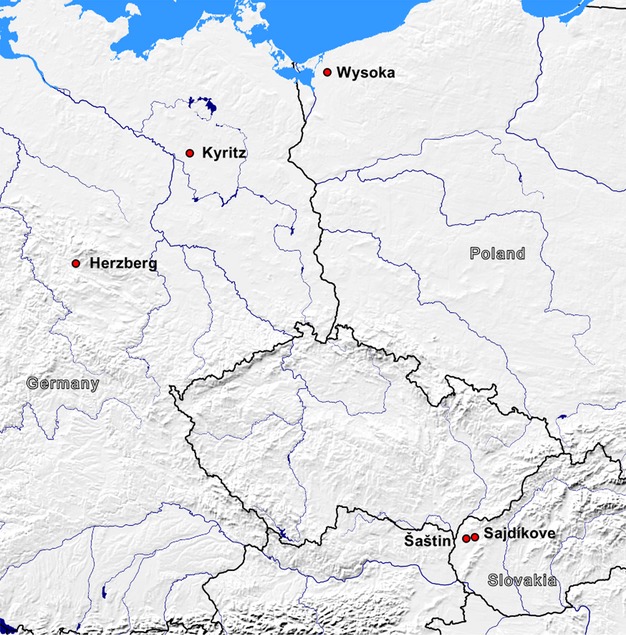
Locations of sampled populations in Germany, Poland, and Slovakia.

We sampled frogs in five populations from three European countries (Fig. 1). In Poland, frogs were caught from two ponds located near Wysoka Kamieńska (53°49′18″N, 14°50′38″E, in this study referred to as Wysoka). In Germany, they originated from one pond situated 2 km south of the village of Herzberg am Harz (51°37′37″N, 10°21′15″E, Herzberg), and from the village pond of Schönermark, near Kyritz (52°54′08″N, 12°19′16″E, Kyritz). In Slovakia, we sampled from two ponds close to the village of Šajdíkove Humence (48°38′34″N, 17°16′54″E, Šajdíkove) and from two ponds located in the village of Šaštín-Stráže (48°37′55″N, 17°08′40″E, Šaštín). Maximum distances between the five populations were 580 km in north–south and 470 km in east–west direction.

Frogs were collected by hand at night using a flashlight. They were identified for sex and taxon on the spot according to phenotypic characteristics (Berger 1988b; Plötner 2005). In order to distinguish diploid from triploid hybrids, we took blood smears and measured erythrocyte lengths and widths under the microscope; in *Pelophylax*, triploid erythrocytes are significantly larger than diploid ones (Berger 1988a; Vinogradov et al. 1990). All frogs were toe clipped for subsequent microsatellite DNA analyses in order to confirm the taxon identification and analyze genotype composition in the total sample. Thereafter, most frogs were released back into the pond of origin; but a few diploid and triploid hybrids were kept for crossing experiments in the laboratory. They were selected on the basis of their size, health, and, in females, signs of gravity. These kept frogs were individually marked with transponders (ID-162, AEG), separated by sex and assumed genotype, and transported to the University of Zurich in cloth bags filled with rubber sponges. During transport, the bags were showered daily with fresh water. All frogs survived the journey.

### Microsatellite analysis

Precise genotype identification of the frogs sampled on site, of the frogs used as parents, and of the offspring resulting from the crosses was achieved through microsatellite analysis. We used a piece of the tailfin (tadpoles) and a fingertip (adults and metamorphs), respectively, as source material. DNA extraction and purification were performed using a Biosprint 96 DNA Blood Kit (Qiagen, Hombrechtikon, Switzerland) in combination with the Biosprint 96 workstation following the supplier's protocol. The purified DNA was subjected to polymerase chain reaction (PCR) runs with four primer mixes involving a total of 18 microsatellites primer pairs:

- Primer Mix 1A: CA1b6, Ga1a19 redesigned (Arioli et al. 2010), RlCA1b5, RlCA5 (Garner et al. 2000), Rrid064A (Christiansen and Reyer 2009).
- Primer Mix 1B: Re2CAGA3 (Arioli et al. 2010), Res16, Res20 (Zeisset et al. 2000), RlCA2a34 (Christiansen and Reyer 2009).
- Primer Mix 2A: ReGA1a23, Rrid169A, Rrid059A redesigned (Christiansen and Reyer 2009), Res22 (Zeisset et al. 2000), Rrid013A (Hotz et al. 2001).
- Primer Mix 2B: Re1Caga10 (Arioli et al. 2010), RlCA18 (Garner et al. 2000), RlCA1a27, Rrid135A (Christiansen and Reyer 2009).

Details on PCR protocols are given by Christiansen (2009) and Christiansen and Reyer (2009, 2011). PCR products were run for fragment length analysis on an Applied Biosystems 3730 Avant capillary sequencer with internal size standard (GeneScan-500 LIZ), and the alleles were scored with the Genemapper software v3.7 (Applied Biosystems, Zug, Switzerland).

Loci Res20, RlCA2a34, ReGa1a23, RlCA1a27, and RlCA18 were species specific for *P. lessonae*, whereas loci Rrid064A, Re2CAGA3, Res22, Re1CAGA10, and Rrid135A were species specific for *P. ridibundus*. The other eight microsatellite loci amplified in both L and R genomes. For these loci species-specificities of the alleles were known from previous studies (Christiansen 2005, 2009; Arioli et al. 2010; N. B. M. Pruvost unpubl. data). Four microsatellite loci (CA1b6, RlCA1b5, Ga1a19 redesigned, and Res16) showed a dosage effect allowing us to determine the ploidy of hybrids by comparing the height of the peaks (Christiansen 2005). The sum and congruence of the 18 microsatellites markers allowed the identification of the consensus genotype of each specimen.

### Population genetics analyses

Because of the hybridogenetic mode of genome transmission which inhibits recombination between the *P. lessonae* (L) and *P. ridibundus* (R) genomes, all analyses were performed for each genome separately. Prior to analyses we tested the microsatellite dataset for the presence of null alleles in both genomes using the software Micro-Checker version 2.2.3 (Van Oosterhout et al. 2004). Because the procedure implemented in Micro-Checker requires diploid data, we could apply this method only to the specimens of the two parental species and to triploid hybrids for the genome present in double copy. For haploid parental genomes, that is, single-copy genomes of triploids and both genomes in diploids, the search for null alleles was done by simple examination of the data. When even after two to three reruns of PCR, no allele was detected, this was taken as an indication for the presence of a null allele. Null alleles were detected in two loci that amplify for both genomes, namely, RlCA5 and Res16. In addition, loci RlCA2a34, ReGA1a23, and Rrid169A showed the presence of null alleles in the R genome, whereas locus Re1CAGA10 betrayed a null allele in the L genome. After excluding these loci from further analyses, we could use the following 10 loci for our calculations: CA1b6, RlCA1b5, Ga1a19 redesigned, Rrid013A and Rrid059 redesigned for both genomes, together with Res20, RlCA2a34, ReGA1a23, RlCA1a27, and RlCA18 for the L genome only, and with Rrid064A, Re2CAGA3, Res22, Re1CAGA10, and Rrid135A for the R genome only.

We investigated population structure by calculating the allelic diversity corrected for sample size (H_e_, expected heterozygosity, according to Nei 1978) and the fixation index (*F*_ST_, according to Weir and Cockerham 1984) using the software SPAGeDi version 1.3 (Hardy and Vekemans 2002) which allows the combination of multiple ploidy levels in the same analysis. Again, because of the independence of the two parental genomes, expected heterozygosity was calculated separately for the L genome (H_eL_) and for the R genomes (H_eR_) for each frog genomotype in each of the studied populations. In order to investigate, how similar, respectively different gene pools are, we compared allelic diversity values between pairs of gene pools of different frog types, by applying two-tailed paired *t*-tests to the values for each locus. We also run nonparametric Wilcoxon signed-rank test which gave the same results. For comparisons between more than two types of frogs within a population we used analyses of variance with H_e_ as dependant variable and loci as fixed effect.

In order to estimate the genetic distances between each genetic pool of different frog types in each population, we calculated pairwise *F*_ST_ values separately for the L genomes of the LL, LR, LLR, and LRR frogs and for the R genomes of the LR, LLR, LRR, and RR frogs, respectively. P values for these *F*_ST_ were obtained by running permutation test with 10,000 iterations. Concerning the interpretation of these values we followed the qualitative guideline proposed by Wright (1978): 0 ≤ *F*_ST_ < 0.05 indicate little genetic differentiation, 0.05 ≤ *F*_ST_ < 0.15 moderate, 0.15 ≤ *F*_ST_ < 0.25 great, and 0.25 ≤ *F*_ST_ very great genetic differentiation.

All statistical tests were run using the program R (version 2.15.1, R Development Core Team 2012).

### Crossing design

In order to determine the type of gamete produced by a given hybrid and to avoid the masking effect of potential genetic incompatibilities between hybrid genomes, we crossed each frog with at least one specimen of each parental species (*P. lessonae* and *P. ridibundus*) and with one other hybrid.

We originally had planned to cross three hybrids of each genomotype from the five populations, but due to insufficient egg numbers in some females and/or failed fertilization through sperm of some males we could not systematically do this (see Table 1). For the same lack of gametes, we also did not perform crosses between parental males and females; but parental offspring resulting from such combinations are not relevant for our questions anyway.

**Table 1:** Population composition, in term of number of frogs caught and number of frogs crossed per genomotypes, for two mixed population (M) where diploid hybrids occur in sympatry with a parental species and three all-hybrid populations consisting of diploid and triploid hybrids

Population	Genomotype									

	LLR		LR		LRR		LL		RR	
				
	♀	♂	♀	♂	♀	♂	♀	♂	♀	♂
Herzberg (M)										
Caught	–	–	6	19	–	–	–	10	–	25
Crossed	–	–	3	3	–	–	–	x	–	x
Šaštin (M)										
Caught	–	–	43	27	–	–	1	27	13	15
Crossed	–	–	5	5	–	–	x	4	2	3
Šajdíkove (H)										
Caught	–	91	30	2	–	–	–	–	–	–
Crossed	–	14	5	1	–	–	–	–	–	–
Kyritz (H)										
Caught	7	19	34	25	24	12	–	1	–	–
Crossed	2	3	3	3	3	3	–	x	–	–
Wysoka (H)										
Caught	3	14	17	10	7	6	–	–	–	–
Crossed	x	2	2	5	1	1	–	–	–	–

Some of the parental species specimens used in crosses came from other populations and are not listed here. **–**, absence of frogs of the respective type; x, frog types which were present in the population but not crossed.

### Artificial crossing procedure

Crosses were performed following the artificial fertilization procedure by Berger et al. (1994) with minor modifications. Ovulation stimulation was triggered by the injection of a solution of LHRH fish hormone (Bachem H-7525, Bubendorf, Switzerland) at 2 mg in 100 mL Holtfreter's solution. We injected 100 μL per 10 g of body mass. After about 24 h, when females were ready for laying eggs, males were euthanized in a buffered (pH 7) MS-222 solution (Sigma A-5040, St. Gallen, Switzerland) at 2 mg/L and their testes were removed, sliced, and crushed in a Petri dish with aged tap water. Eggs were gently stripped into this sperm suspension, where they remained for about 2–3 min. After this period, the suspension was rinsed into a new Petri dish where eggs of another female were added. This protocol allows the use of the same sperm solution to fertilize eggs from different females and to fertilize eggs of the same female with sperm from different males. Eggs were covered with aged tap water and checked for fertilization success, identified by a rotation that moves the black animal hemisphere to the top within the next 30–60 min. The next day, all eggs were transferred to 6-L containers with 1–2 cm of water. After 2 days, unfertilized eggs, egg jelly, and/or aborted embryos were carefully removed every 2 days to avoid bacterial and fungal development. After about 15 days embryos started to reach free swimming stage (stage 25, Gosner 1960) and were euthanized using the MS-222 buffered solution cited above. The offspring of a few crosses were used for other experiments (Pruvost et al. 2013), but their genotypic data could also be use for our purpose. All studied offspring reached at least stage 25.

### Gamete production determination

Originally, we had planned to genotype a minimum of 35 offspring for each cross. However, due to limited egg availability, low fertilization success, and/or inviable offspring, probably resulting from genetic incompatibilities, for some crosses this goal was not reached, whereas for others more than 35 offspring could be genotyped (see Appendix S1). After identifying the offspring genotypes, and knowing the genotypes of their mothers and fathers, we could determine the types and relative numbers of gametes produced by each of the two parents. As each parent frog was used in more than one cross, we summed up the results obtained from all crosses involving this frog. Potential problems caused by parental infertility or genetic incompatibilities which may mask the actual gamete production would have been revealed by a differential gamete production patterns among crosses involving the same frog. If, for instance, a frog produced no viable offspring with any of the individuals it was crossed to, this would indicate infertility, whereas failure in only one or the other cross suggests genetic incompatibility with the particular partner. However, neither was found.

## Results

### Population composition

Microsatellite analysis allowed us to determine the genomotypes of 488 adult frogs sampled in the five populations. Population compositions in terms of taxa and ploidy are shown in Table 1. In two populations (Herzberg, Šaštin) – from now on called “mixed populations” – diploid hybrid males and females occurred in sympatry with both parental species, whereas in the other three populations only hybrids were found (“all-hybrid populations”), with the exception of one LL individual in Kyritz. In Šaštin, individuals of the two parental species existed in both sexes, but in Herzberg only males were captured.

The three all-hybrid populations also differed in their composition. In Kyritz and Wysoka, we caught all three possible genomotypes (LR, LLR, and LRR) in both sexes, but in Šajdíkove LRR was absent, LLR consisted exclusively of males and LR almost only of females (with the exception of two diploid males). Given the large number of frogs caught in this population (*n* = 123, Table 1), this genotype and sex bias is highly unlikely to have resulted from chance effects in a small sample.

In Šajdíkove, microsatellite dosage effect revealed the presence of one tetraploid male (LLRR) possessing the same double L genome as the triploids in addition to a double R genome completely homozygote for the studied loci.

### Populations genetic structure

#### Allelic diversity

The mean allelic diversity for the 10 loci considered is shown in Table 2 for each genome separately and detailed by loci in Appendix S2. In the two mixed populations, L genome allelic diversity (H_eL_) did not differ between LR hybrids and parental LL (Šaštin: mean difference = 0.007 ± 0.032, *t*_(9)_ = 0.215, *P* = 0.834; Herzberg: m.d. = 0.073 ± 0.070, *t*_(9)_ = 1.045, *P* = 0.323), nor did R genome allelic diversity (H_eR_) differ between LR and parental RR in Herzberg (m.d. = 0.015 ± 0.043, *t*_(9)_ = 0.347, *P* = 0.736); but in Šaštin it did (m.d. = 0.240 ± 0.050, *t*_(9)_ = 4.799, *P* = 0.001), with the *P. ridibundus* parental species showing a higher allelic diversity (H_eR_ = 0.625) than the LR hybrids (H_eR_ = 0.384).

**Table 2:** Mean allelic diversity corrected for sample size, Nei 1978 (H_e_) for *P. lessonae* genomes (H_eL_) and *P. ridibundus* genomes (H_eR_) in the different frog types (LL, LLR, LR, LRR, and RR)

Population	H_eL_				H_eR_			
	
	LL	LLR	LR	LRR	LLR	LR	LRR	RR
Herzberg	0.441 (10)	–	0.368 (25)	–	–	0.380 (25)	–	0.395 (25)
Šaštin	0.428 (28)	–	0.421 (70)	–	–	0.384 (70)	–	0.625 (28)
Šajdíkove	–	0.201 (91)	0.452 (32)	–	0.432 (91)	0.402 (32)	–	–
Kyritz	–	0.321 (26)	0.300 (59)	0.284 (36)	0.358 (26)	0.404 (59)	0.401 (36)	–
Wysoka	–	0.240 (17)	0.221 (27)	0.212 (13)	0.512 (17)	0.554 (27)	0.609 (13)	–

Sample size is given in brackets.

With respect to the all-hybrid populations, analyses of variance did not detect any differences in both H_eL_ and H_eR_ between diploid (LR) and triploid (LLR, LRR) hybrids in Wysoka and Kyritz where all three genomotypes occur (Table 2). In contrast, in Šajdíkove, with (mostly) LR females and only LLR males, H_eL_ values differ greatly between diploids and triploids (m.d. = 0.251 ± 0.080, *t*_(9)_ = 3.130, *P* = 0.012) with diploid hybrids showing a higher allelic diversity than the triploid LLR males. While H_eR_ values do not (m.d. = 0.029 ± 0.016, *t*_(9)_ = −1.862, *P* = 0.095). In this population the allelic composition of all expressed loci of the double L genome of the triploid males is exactly the same among all specimens, meaning that all LL genomes in all LLR males are genetically identical.

#### Population differentiation

The overall genetic differentiations (represented by global *F*_ST_ values) shows substantial and highly significant differentiation among populations for both genomes, assigning 43.59% of the variation in the L genome (global *F*_ST_ = 0.436, *P* < 0.001) and 25.42% in the R genome (global *F*_ST_ = 0.254, *P* < 0.001) to interpopulation differences.

The pairwise *F*_ST_ values between each frog genomotype in each population are given in Table 3. In the two mixed populations, there is little differentiation between LR and LL in the L genome (Šaštin: *F*_ST_ = 0.028; Herzberg: *F*_ST_ = 0.024) and little to moderate differentiation between LR and RR in the R genome (Herzberg: *F*_ST_ = 0.033; Šaštin: *F*_ST_ = 0.138). Among the all-hybrid populations, differentiation is low for both genomes within Wysoka and Kyritz, where all three hybrid types occur (all *F*_ST_ ≤ 0.041) In Šajdíkove, with only two hybrid types differentiation between LLR males and mostly LR females is also low for the R genomes (*F*_ST_ = 0.008), but very high for the L genomes (*F*_ST_ = 0.517).

**Table 3:** Pairwise *F*_ST_ values using Weir and Cockerham (1984) calculation

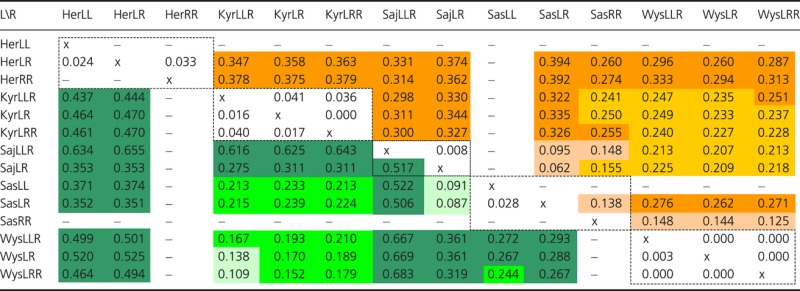

Values for the R genomes are above the diagonal and values for the L genomes under it. 0 ≤ *F*_ST_ < 0.05 indicates little genetic differentiation (uncolored boxes), 0.05 ≤ *F*_ST_ < 0.15 moderate (light green for L and light orange for R), 0.15 ≤ *F*_ST_ < 0.25 great (green for L and orange for R), and 0.25 ≤ *F*_ST_ very great genetic differentiation (dark green for L and dark orange for R) (Wright 1978). x, no values calculated inside the same group of frogs. -, no value calculated because of the absence of one specific genome in the parental species.

### Gamete production

We performed a total of 198 crosses involving 64 *P. esculentus* (35 LR, 21 LLR, and 8 LRR), 18 *P. lessonae*, and 15 *P. ridibundus*. We genotyped the 97 adults crossed and 4675 tadpoles resulting from these crosses. The results of the gametes produced are presented in Appendix S1.

In two populations we encountered problems which resulted in low offspring numbers or even no offspring at all (for details see column *N* off. in Appendix S1). These problems resulted from lack of sufficient mature eggs in some females, sexual immaturity of few males, and a combination of the two causes. Overall, however, we managed to analyze the proportions of gamete types produced by every hybrid type in each population, except for the only LLR males from Wysoka (see Appendix S1).

In the mixed populations of Herzberg and Šaštin, hybrid LR frogs of both sexes always produced haploid gametes with a clonally transmitted R genome. Among the all-hybrid populations, the pattern was more diverse.

In Kyritz, as well as in Wysoka, diploid males also exclusively produced haploid gametes with a clonally transmitted R genome, but all diploid females produced diploid LR gametes, with the exception of one female from Kyritz (WFB014-20) which produced equal numbers of R and LR eggs. Among the triploids, the prevailing pattern was the production of haploid gametes with a recombined genome of the type that is present in two copies, that is, L in LLR and R in LRR. Without any exception this was true for all LRR of both sexes and all LLR males, whereas in LLR females it applied to only 89% of the eggs. The remaining 11% contained diploid clonally transmitted LL genomes.

In Šajdíkove, triploid males always produced diploid gametes, which clonally transmit two L genomes. The microsatellite genotyping revealed that the LL multilocus genotype of all these frogs is exactly the same in all adults males caught on site and in all the offspring produced by our crosses. The diploid males and females from this population produced only clonal haploid R gametes. The general pattern of gamete production is given in Table 4.

**Table 4:** Gamete production of the different genomotypes of hybrids and inferred breeding systems in the five studied populations

Population	Genomotype						Inferred breeding system

	LLR		LR		LRR		
		
	Female	Male	Female	Male	Female	Male	
Herzberg	–	–	R	R	–	–	L-E
Šaštin	–	–	R	R	–	–	L-E
Šajdíkove	–	LL	R	R	–	–	Modified L-E
Kyritz	L (LL)	L	LR (R)	R	R	R	E-E
Wysoka	L	L	LR	R	R	R	E-E

Gamete types in parentheses are produced in small proportions.

## Discussion

The gamete production patterns found in this study confirm the expected mixture of clonally and recombining genomes traveling between different frog genomotypes. In combination with H_e_ and pairwise *F*_ST_ values, which allow estimating levels of genetic differentiation between gene pools of all frog genomotypes, we can describe the genetic interactions happening in the different populations and link them to known breeding system types occurring in water frogs. In the following paragraphs, we propose an evolutionary scenario for the appearance and maintenance of these systems.

### Gamete production pattern

**Figure 2:**
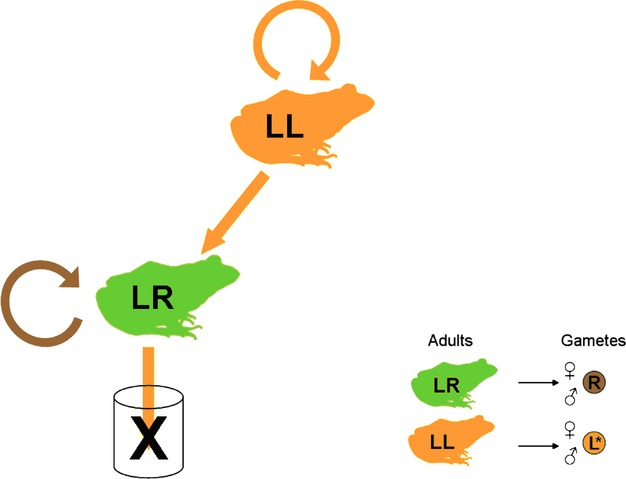
“LE-system” scheme showing the transmission of the L (orange arrow) and of the R (brown arrow) genomes and the gamete production pattern of the different frogs genomotypes. The * in the gametes indicates recombining genomes.

Diploid hybrids always transmitted clonal genomes, either haploid R or diploid LR. The production of haploid gametes with clonal R genomes is in accordance with the hemiclonal transmission mode expected in LE-systems (Fig. 2), where the previously excluded L genome is regained by mating with *P. lessonae*, and thus hybridity restored. In contrast, the production of diploid gametes carrying clonal copies of the entire LR maternal genome is a feature expected of diploid females from all-hybrid populations of the EE-system (Fig. 4) (Christiansen 2009). Here, the L and R genomes that are necessary for maintaining all three hybrid types in the population (LR, LLR, and LRR) are provided by triploids that produce recombined haploid gametes of the type that is present in two copies (Christiansen and Reyer 2009; Morishima et al. 2008). With the slight modification in two Kyritz LLR females which produced a few diploid gametes containing their two L genomes, this was the pattern found in triploid frogs from Kyritz and Wysoka.

**Figure 3:**
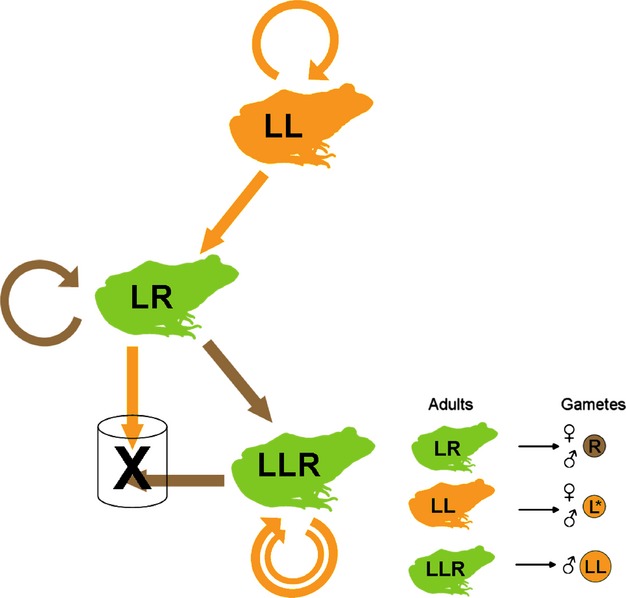
“Modified LE-system” scheme showing the transmission of the L (orange arrow) and of the R (brown arrow) genomes and the gamete production pattern of the different frog genomotypes. The * in the gametes indicates recombining genomes.

While these results confirm those from previous studies, the gamete production pattern in LLR males from Šajdíkove, with clonally produced sperm containing their double L genomes, suggests a previously not described “modified LE-system” (Fig. 3). Below, we discuss the three breeding systems in more detail.

### Breeding systems

#### LE-systems

In typical LE-systems, diploid hybrids discard the L genome prior to meiosis, produce clonal R gametes, and restore hybridity by mating with the sexual *P. lessonae* parental species which provides gametes with a new recombined L genome. In such systems, the hybrids are sexual parasites of the *P. lessonae* parental species and act as a sink for the host's L genome (Schmidt 1993; Joly 2001; Lehtonen et al. 2013). In our study, this system is represented by the populations in Herzberg and Šaštin (Fig. 2).

In Šaštin, allelic diversity in the R genome (H_eR_) is lower in LR hybrids (with no recombination) than in RR frogs (with recombination), and there is moderate genetic differentiation between LR and RR frogs (*F*_ST_ = 0.138). In contrast, allelic diversity in the L genome (H_eL_) is equally high for LR and LL frogs and genetic differentiation between their genomes is low (*F*_ST_ = 0.028) (Tables 2). This is in line with the genome transmission mode in LE-systems: clonal R versus sexual L.

In Herzberg, the situation appears a bit different regarding the role of the sympatric *P. ridibundus* frogs. The relatively low genetic differentiation between LR and RR frogs in the R genome and the quite similar values of gene diversity are a hint for close interactions between the two gene pools. In both populations, however, allelic diversity and genetic differences may not only reflect the genome transmission mode but also be influenced by the number of original primary hybridization which will affect diversity in the clonal R genome. Unfortunately, empirical data about primary hybridizations are lacking for both populations.

#### Modified LE-system

In Šajdíkove, the gamete production pattern of the diploid hybrids is the same as the one occurring in LE-systems, but this population also contains triploid hybrid LLR males, which always produce diploid LL gametes containing identical copies of the two same genomes. This mode of transmission is clearly reflected by the population genetic indices (Fig. 3):

- First, the *F*_ST_ value estimating the differentiation of the L genome between LLR and LR frogs within Šajdíkove is very high.
- Second, allelic diversity in the L genomes is significantly lower in LLR frogs (H_eL_ = 0.201) which receive a clonal LL genome than in LR frogs (H_eL_ = 0.452), where the value is similar to those of LL and LR frogs from LE-systems (Table 2). This suggests that diploid hybrids in Šajdíkove received recombined L genomes. Another, not mutually exclusive, explanation of the higher allelic diversity in L genomes of LR frogs is that new lineages have been produced on multiple occasions.

However, both explanations cannot answer the question where the haploid L genomes (which are required to produce diploid hybrids) originate from. In the sampled ponds, no *P. lessonae* were found. They may occur in ponds nearby.

This hypothesis is consistent with the moderate genetic differentiation values found between the diploid LR from Šajdíkove and both the diploid LR and parental species LL in Šaštin. Also, haploid L gametes may occasionally be produced by diploid LR (as in the RE-system) or by triploid LLR (as in most EE-systems). For this, however, our crossing experiment provided no evidence (see Appendix S1).

The triploid males that transmit their double L genome and mate with diploid LR females producing R eggs sire offspring of their own genomotype. Hence, they form a unique paternal hemiclonal lineage with a frozen double L genome. As these LLR frogs exclude the R genome at gametogenesis, they are acting as a sink for the R genome, which is transmitted by LR frogs that, in turn, are acting as a genetic sink for the L genome (Fig. 3). Given that the L genome of the diploids must come from another source (see above), the triploid males in the population are not essential to the perpetuation of the diploids in the breeding system. They just seem to have found a way to persist by parasitizing the R genomes of the sympatric LR hybrids. In contrast to EE-systems, which could not exist without triploids (see below), LLR males in Šajdíkove can be seen as a mere add-on to the L-E system. We, therefore, decided to name such breeding system as “modified LE-system.” This breeding system type is not restricted to this western Slovak population. Some triploid LLR males carrying the same two genomes (with only a 2 bp difference in one allele of the 18 microsatellite loci) have also been found in populations from the northeastern part of the Czech Republic, 130 km north, in the locality of Borovec (N. B. M. Pruvost, P. Mikulíček, L. Choleva and H.-U. Reyer, unpubl. ms.).

#### EE-systems

**Figure 4:**
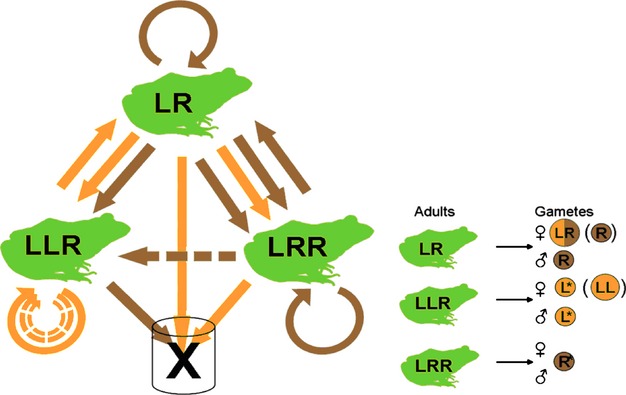
“EE-system” scheme showing the transmission of the L (orange arrow) and of the R (brown arrow) genomes and the gamete production pattern of the different frog genomotypes. Gamete types in parenthesis are produce in low frequency. Dashed arrows represent transmission with low frequency. The * in the gametes indicates recombining genomes.

The gamete production pattern of frogs from Kyritz and Wysoka corresponds to the EE-system that was intensively studied and described for Denmark and southern Sweden by Christiansen and Reyer (2009), Arioli et al. (2010), and Jakob et al. (2010). In such systems, the three different hybrid genomotypes manage to produce all the gamete types needed for their coexistence without requiring the presence of any of the two parental species. Diploid LR eggs are produced by diploid LR females, haploid R sperm by diploid LR males, and recombined haploid L and R gametes by males and females of triploid LLR and LRR, respectively. This genetic functioning is perfectly reflected in the two population genetics parameters we used. In both populations, the gene diversity values for both genomes are in the same range for the three frog genomotypes. Pairwise *F*_ST_ values within populations also demonstrate very little genetic differentiation between the three genomotypes. In such breeding systems all frog genomotypes depend on each other to be produced (Fig. 4):

- LR frogs arise from the combination of L gametes, exclusively produced by LLR frogs, with R gametes produced by LRR specimens, LR males and (in smaller proportion) LR females.
- LLR frogs mainly arise from fertilization of LR eggs produced by LR females with L sperm from males of their own genomotype, or (in smaller proportions) by fusion of R sperm coming from LRR and LR males with LL eggs from females of their own genomotype.
- LRR frogs only arise from the combination of LR eggs from LR females and R sperm produced by LR and LRR males.

Thus, LR and LLR frog types are absolutely necessary to the system in their role as producers of LR and L gametes, respectively, whereas LRR frogs are crucial as producers of R gametes, especially R eggs which only rarely are produced by LR females. Under these conditions, the EE-system would collapse if one of the actors would be removed. As predicted by the model of Som and Reyer (2006), such EE-system can persist under random mating which, indeed, seems to occur. In contrast to hybrid females from LE-systems that prefer *P. lessonae* over *P. esculentus* males (Abt and Reyer 1993; Roesli and Reyer 2000; Engeler and Reyer 2001), females from all-hybrid populations show no preference (Günther and Plötner 1989; Rondinelli 2006). As triploid hybrids recombine the genome they have in double dose (Christiansen and Reyer 2009), they provide genetic diversity equivalent to the one found in the parental species, giving such systems an evolutionary potential comparable to that of sexually reproducing populations.

### Origins and evolutionary potential of systems involving triploid hybrids

The difference in gamete production patterns, leading to the existence of triploid specimens in Wysoka and Kyritz on the one hand and in Šajdíkove on the other strongly suggests a polyphyletic origin of triploid frogs in EE- and modified LE-systems. Both systems may have developed from the most widespread typical LE-system (Fig. 2) because all three systems are identical in that LR males produce clonal haploid R gametes; but then differences arose from the mechanisms that lead to the production triploid individuals: Fusion of LR eggs from LR females with haploid sperm in the EE-system as opposed to fusion of haploid eggs with LL sperm from LLR males in the modified LE-system. The perfect identity of the two L genomes present in triploid LLR males from the modified LE-system suggests that this lineage probably arose from a single event of L genome doubling that generated an array of clones, or even from one single triploid specimen. Unraveling the origin of such frogs would demand a much broader population genetics investigations. However, whatever their origin, the 3n males in this system do not participate in the generation of the two other frog types (LL and LR). They only exploit R genomes from the pool of eggs produced by LR females and use their own double L genome to procreate themselves. They act as a sink for the R genome which already parasitizes the parental species sexual L genome. Thus, in contrast to EE-systems which depend on the presence of triploids, triploids from the modified LE-system could disappear without harming the persistence of the other frog types, thus leaving an intact LE-system behind.

Concerning the EE-systems, the initial step away from the typical LE-system must have been a suppression of L genome exclusion in LR females, resulting in the clonal transmission of LR, rather than R genomes. Once produced, these 2n eggs automatically lead to both types of triploids: Mating with *P. lessonae* males produces LLR offspring and mating with diploid *P. esculentus* hybrids produces LRR offspring. Due to the so-called meiotic hybridogenesis mechanism (Alves et al. 1998; Cunha et al. 2008), LLR frogs are then able to produce recombined haploid L gametes and thus replace *P. lessonae* frogs, whereas LRR frogs can act as haploid R gamete donors and – in case of females – adopt the role previously fulfilled by LR females which now produce diploid LR eggs.

With the *P. lessonae* parental species having lost its essential position in maintaining the system, the hybrids become independent from the parental species, can disperse into environments where *P. lessonae* is absent, and establish all-hybrid populations (EE-system). In combination with differential ecological tolerance leading to a competitive advantage for the hybrid, these populations can be maintained even if later on the parental species also disperses into that habitat. In fact, the better performance of hybrids compared to the parental species under cold conditions offers a possible explanation why the EE-system is widespread in colder region like the north of Europe (Negovetic et al. 2001; Pruvost et al. 2013).

This scenario highlights the high evolutionary potential of this seemingly flawed water frog system. What at first glance appears to be a failure of the typical gamete production pattern can, in situations where its meets favorable ecological condition, lead to completely new and evolutionary significant population types and breeding systems capable of colonizing new geographical ranges. Natural events and/or introduction may have led to some more population types and breeding systems with unusual combinations of different gametes donor types. Therefore, further detailed studies of the European water frog group seem justified and promising. Nevertheless, at least in the case of the EE-systems, our results support Schultz’ (1989) statement “…non-Mendelian forms of hybrid origin have evolved adaptations distinct from parental biotypes and have assumed evolutionary directions that are different and independent of them.”

This insight is also relevant from a conservation point of view. Modern management concepts stress the importance of conserving “evolutionary significant units” (ESUs), that is, populations representing significant adaptive variation; but how these units are to be identified is strongly debated (reviewed by Crandall et al. 2000; Pearman 2001). Hybrids, for instance, are exempt from protection because they do not seem to constitute independent evolutionary lineages (Kraus 1995). This, however, does not hold for parthenogenetic, gynogenetic, and hybridogenetic taxa that are originally of hybrid origin, propagate only the maternally inherited genome, and may carry the potential for speciation via polyploidy. Depending on their genetic distinctiveness, their success in various environments, and the effective size of their populations, they, therefore, may require special protection efforts (Kraus 1995).

## Supporting Information

**Appendix S1.** Gamete production of the crossed frogs. Population, name of the population of origin; Geno., genotype of the parent; Ind. Numb., specimen number; *N* cross, number of crosses involving this frog; *N* off., number of offspring genotyped; Gamete type, genomic composition and ploidy of the gametes produced.

**Appendix S2.** Allelic diversity corrected by sample size (Nei 1978) for each locus in the different frog types, for the L and the R genome, respectively.
