# Supplementary material for: Gamete production patterns, ploidy, and population genetics reveal evolutionary significant units in hybrid water frogs (Pelophylax esculentus)
Source: Ecol Evol. 2013 Jul 30;3(9):2933–46. doi: 10.1002/ece3.687 (PMC3790541; doi:10.1002/ece3.687)
Supplement: Supplementary file 1 [file ece30003-2933-SD1.doc]

# Supplementary material

**Appendix 1:** Gamete production of the crossed frogs. “Population” stands for the name of the population of origin, “Geno.” for the genotype of the parent, “Ind. Numb.” for its specimen number, “N cross” for the number of crosses involving this frog, “N off.” for the number of offspring genotyped and “Gamete type for the genomic composition and ploidy of the gametes produced.

| Population | Geno. | Sex | Ind. numb. | N cross | N off. | Gamete type | | | |
| --- | --- | --- | --- | --- | --- | --- | --- | --- | --- |
| Herzberg | LR | F | WFB021-27 | 3 | 99 | 100 R |  |  |  |
|  |  |  | WFB021-28 | 4 | 103 | 100 R |  |  |  |
|  |  |  | WFB021-29 | 3 | 108 | 100 R |  |  |  |
|  |  | M | WFB021-12 | 5 | 73 | 100 R |  |  |  |
|  |  |  | WFB021-21 | 3 | 40 | 100 R |  |  |  |
|  |  |  | WFB021-22 | 3 | 57 | 100 R |  |  |  |
| Kyritz | LLR | F | WFB014-21 | 3 | 74 |  | 91.9 L |  | 8.1 LL |
|  |  |  | WFB014-62 | 11 | 356 |  | 86.2 L |  | 13.8 LL |
|  |  | M | WFB014-55 | 4 | 173 |  | 100 L |  |  |
|  |  |  | WFB014-56 | 6 | 208 |  | 100 L |  |  |
|  |  |  | WFB014-59 | 7 | 143 |  | 100 L |  |  |
|  | LR | F | WFB014-20 | 7 | 12 | 50 R |  | 50 LR |  |
|  |  |  | WFB014-25 | 10 | 468 |  |  | 100 LR |  |
|  |  |  | WFB014-63 | 7 | 158 |  |  | 100 LR |  |
|  |  | M | WFB014-05 | 7 | 303 | 100 R |  |  |  |
|  |  |  | WFB014-14 | 4 | 79 | 100 R |  |  |  |
|  |  |  | WFB014-48 | 6 | 156 | 100 R |  |  |  |
|  | LRR | F | WFB014-24 | 9 | 284 | 100 R |  |  |  |
|  |  |  | WFB014-26 | 7 | 272 | 100 R |  |  |  |
|  |  |  | WFB014-67 | 7 | 264 | 100 R |  |  |  |
|  |  | M | WFB014-11 | 6 | 161 | 100 R |  |  |  |
|  |  |  | WFB014-49 | 7 | 274 | 100 R |  |  |  |
|  |  |  | WFB014-58 | 4 | 143 | 100 R |  |  |  |
| Šajdíkove | LLR | M | WFB007-93 | 4 | 86 |  |  |  | 100 LL |
|  |  |  | WFB008-14 | 3 | 93 |  |  |  | 100 LL |
|  |  |  | WFB015-13 | 4 | 10 |  |  |  | 100 LL |
|  |  |  | WFB015-55 | 5 | 178 |  |  |  | 100 LL |
|  |  |  | WFB015-56 | 2 | 11 |  |  |  | 100 LL |
|  |  |  | WFB015-57 | 2 | 25 |  |  |  | 100 LL |
|  |  |  | WFB021-16 | 3 | 3 |  |  |  | 100 LL |
|  |  |  | WFB021-17 | 3 | 21 |  |  |  | 100 LL |
|  |  |  | WFB021-18 | 3 | 16 |  |  |  | 100 LL |
|  |  |  | WFB008-16 | 2 | 0 |  |  |  |  |
|  |  |  | WFB015-09 | 6 | 0 |  |  |  |  |
|  |  |  | WFB015-10 | 4 | 0 |  |  |  |  |
|  |  |  | WFB021-19 | 2 | 0 |  |  |  |  |
|  |  |  | WFB016-42 | 7 | 0 |  |  |  |  |
|  | LR | F | WFB021-24 | 5 | 104 | 100 R |  |  |  |
|  |  |  | WFB021-30 | 3 | 96 | 100 R |  |  |  |
|  |  |  | WFB007-91 | 2 | 30 | 100 R |  |  |  |
|  |  |  | WFB021-25 | 1 | 0 |  |  |  |  |
|  |  |  | WFB021-26 | 1 | 0 |  |  |  |  |
|  |  | M | WFB007-90 | 2 | 8 | 100 R |  |  |  |
| Šaštin | LR | F | WFB007-33 | 1 | 8 | 100 R |  |  |  |
|  |  |  | WFB007-35 | 1 | 12 | 100 R |  |  |  |
|  |  |  | WFB007-37 | 4 | 141 | 100 R |  |  |  |
|  |  |  | WFB015-72 | 8 | 283 | 100 R |  |  |  |
|  |  |  | WFB015-73 | 7 | 161 | 100 R |  |  |  |
|  |  | M | WFB007-52 | 4 | 101 | 100 R |  |  |  |
|  |  |  | WFB007-54 | 5 | 79 | 100 R |  |  |  |
|  |  |  | WFB015-03 | 6 | 84 | 100 R |  |  |  |
|  |  |  | WFB015-04 | 4 | 133 | 100 R |  |  |  |
|  |  |  | WFB015-06 | 7 | 254 | 100 R |  |  |  |
| Wysoka | LLR | M | WFB003-02 | 2 | 66 |  | 100 L |  |  |
|  |  |  | WFB003-04 | 1 | 29 |  | 100 L |  |  |
|  | LR | F | WFB002-80 | 4 | 22 |  |  | 100 LR |  |
|  |  |  | WFB002-81 | 2 | 45 |  |  | 100 LR |  |
|  |  | M | WFB002-88 | 2 | 64 | 100 R |  |  |  |
|  |  |  | WFB002-92 | 2 | 66 | 100 R |  |  |  |
|  |  |  | WFB002-93 | 2 | 3 | 100 R |  |  |  |
|  |  |  | WFB002-94 | 1 | 1 | 100 R |  |  |  |
|  |  |  | WFB003-06 | 1 | 0 |  |  |  |  |
|  | LRR | F | WFB002-74 | 4 | 6 | 100 R |  |  |  |
|  |  | M | WFB002-91 | 1 | 0 |  |  |  |  |

Appendix 2: Allelic diversity corrected by sample size (Nei 1978) for each locus in the different frog types, for the L and the R genome respectively.

| Population | | | All | Herzberg | | | Kyritz | | | Sajdikove | | Sastin | | | Wysoka | | |
| --- | --- | --- | --- | --- | --- | --- | --- | --- | --- | --- | --- | --- | --- | --- | --- | --- | --- |
| Genomotype | | | All | LL | LR | RR | LLR | LR | LRR | LLR | LR | LL | LR | RR | LLR | LR | LRR |
| N | | | 434 L  449 R | 10 | 25 | 25 | 26 | 59 | 36 | 91 | 32 | 28 | 70 | 28 | 17 | 27 | 13 |
| L genome | Locus | CA1b6 | 0.415 | 0.479 | 0.513 | - | 0.000 | 0.000 | 0.000 | 0.503 | 0.498 | 0.486 | 0.448 | - | 0.000 | 0.000 | 0.000 |
|  |  | RICA1b5 | 0.136 | 0.337 | 0.347 | - | 0.000 | 0.000 | 0.000 | 0.000 | 0.446 | 0.308 | 0.248 | - | 0.000 | 0.000 | 0.000 |
|  |  | Ga1a19red | 0.084 | 0.505 | 0.520 | - | 0.000 | 0.000 | 0.000 | 0.000 | 0.000 | 0.000 | 0.000 | - | 0.000 | 0.000 | 0.000 |
|  |  | Res20 | 0.685 | 0.668 | 0.417 | - | 0.652 | 0.521 | 0.348 | 0.000 | 0.665 | 0.203 | 0.472 | - | 0.000 | 0.000 | 0.000 |
|  |  | RICA2a34 | 0.838 | 0.468 | 0.587 | - | 0.741 | 0.831 | 0.833 | 0.503 | 0.665 | 0.785 | 0.755 | - | 0.212 | 0.268 | 0.000 |
|  |  | ReGa1a23 | 0.861 | 0.747 | 0.720 | - | 0.835 | 0.842 | 0.746 | 0.503 | 0.843 | 0.868 | 0.851 | - | 0.711 | 0.568 | 0.500 |
|  |  | Rrid013A | 0.537 | 0.633 | 0.000 | - | 0.266 | 0.303 | 0.246 | 0.000 | 0.121 | 0.405 | 0.380 | - | 0.148 | 0.268 | 0.000 |
|  |  | Rrid059Ared | 0.034 | 0.000 | 0.000 | - | 0.000 | 0.000 | 0.000 | 0.000 | 0.000 | 0.000 | 0.000 | - | 0.369 | 0.268 | 0.500 |
|  |  | RlCa1A27 | 0.772 | 0.000 | 0.080 | - | 0.713 | 0.505 | 0.610 | 0.000 | 0.623 | 0.597 | 0.478 | - | 0.649 | 0.690 | 0.833 |
|  |  | RICA18 | 0.658 | 0.568 | 0.493 | - | 0.000 | 0.000 | 0.056 | 0.503 | 0.663 | 0.634 | 0.581 | - | 0.308 | 0.143 | 0.282 |
|  | Mean | | 0.502 | 0.441 | 0.368 | - | 0.321 | 0.300 | 0.284 | 0.201 | 0.452 | 0.428 | 0.421 | - | 0.240 | 0.221 | 0.212 |
|  | Standard Error | | 0.101 | 0.082 | 0.081 | - | 0.117 | 0.111 | 0.105 | 0.082 | 0.096 | 0.096 | 0.089 | - | 0.085 | 0.078 | 0.096 |
|  |  |  |  |  |  |  |  |  |  |  |  |  |  |  |  |  |  |
| R genome | Locus | CA1b6 | 0.685 | - | 0.453 | 0.497 | 0.492 | 0.439 | 0.453 | 0.608 | 0.502 |  | 0.665 | 0.795 | 0.331 | 0.336 | 0.563 |
|  |  | RICA1b5 | 0.237 | - | 0.220 | 0.040 | 0.271 | 0.402 | 0.351 | 0.000 | 0.000 | - | 0.000 | 0.346 | 0.485 | 0.484 | 0.492 |
|  |  | Ga1a19red | 0.500 | - | 0.280 | 0.078 | 0.077 | 0.345 | 0.263 | 0.452 | 0.353 | - | 0.162 | 0.638 | 0.471 | 0.647 | 0.668 |
|  |  | Rrid064A | 0.644 | - | 0.513 | 0.509 | 0.271 | 0.129 | 0.108 | 0.602 | 0.554 | - | 0.111 | 0.349 | 0.603 | 0.711 | 0.750 |
|  |  | Re2CAGA3 | 0.880 | - | 0.663 | 0.691 | 0.754 | 0.712 | 0.691 | 0.788 | 0.805 | - | 0.712 | 0.768 | 0.757 | 0.852 | 0.855 |
|  |  | Res22 | 0.545 | - | 0.280 | 0.393 | 0.077 | 0.230 | 0.309 | 0.510 | 0.444 | - | 0.487 | 0.811 | 0.500 | 0.490 | 0.607 |
|  |  | Rrid013A | 0.077 | - | 0.280 | 0.458 | 0.000 | 0.000 | 0.000 | 0.000 | 0.000 | - | 0.000 | 0.280 | 0.000 | 0.000 | 0.000 |
|  |  | Rrid059Ared | 0.475 | - | 0.347 | 0.509 | 0.409 | 0.471 | 0.477 | 0.022 | 0.063 | - | 0.412 | 0.800 | 0.539 | 0.563 | 0.607 |
|  |  | Re1CAGA10 | 0.825 | - | 0.380 | 0.274 | 0.745 | 0.723 | 0.714 | 0.691 | 0.655 | - | 0.576 | 0.791 | 0.875 | 0.899 | 0.865 |
|  |  | Rrid135A | 0.726 | - | 0.380 | 0.497 | 0.480 | 0.588 | 0.643 | 0.644 | 0.647 | - | 0.716 | 0.670 | 0.564 | 0.563 | 0.679 |
|  | Mean | | 0.559 | - | 0.380 | 0.395 | 0.358 | 0.404 | 0.401 | 0.432 | 0.402 | - | 0.384 | 0.625 | 0.512 | 0.554 | 0.609 |
|  | Standard Error | | 0.080 | - | 0.042 | 0.065 | 0.085 | 0.075 | 0.076 | 0.097 | 0.092 | - | 0.092 | 0.068 | 0.075 | 0.082 | 0.078 |
